# Experimental Evaluation of Tensile Performance of Aluminate Cement Composite Reinforced with Weft Knitted Fabrics as a Function of Curing Temperature

**DOI:** 10.3390/polym13244385

**Published:** 2021-12-14

**Authors:** Bentolhoda Adosi, Seyed Abbas Mirjalili, Mostafa Adresi, Jean-Marc Tulliani, Paola Antonaci

**Affiliations:** 1 Department of Textile Engineering, Yazd University, Yazd P.O. Box 8915818411, Iran; adosi1371@stu.yazd.ac.ir; 2 Department of Civil Engineering, Shahid Rajaee Teacher Training University, Tehran P.O. Box 16785-163, Iran; 3 Department of Applied Science, Politecnico di Torino, P.O. Box 00518460019, 10129 Turin, Italy; 4 Department of Structural, Geotechnical and Building Engineering, Politecnico di Torino, P.O. Box 00518460019, 10129 Turin, Italy

**Keywords:** calcium aluminate cement, weft-knitted 3D fabric, mortar, fracture energy

## Abstract

Cement composites (CC) are among the composites most widely used in the construction industry, such as a durable waterproof and fire-resistant concrete layer, slope protection, and application in retaining wall structures. The use of 3D fabric embedded in the cement media can improve the mechanical properties of the composites. The use of calcium aluminate cement (CAC) can accelerate the production process of the CC and further contribute to improving the mechanical properties of the cement media. The purpose of this study is to promote the use of these cementitious composites by deepening the knowledge of their tensile properties and investigating the factors that may affect them. Therefore, 270 specimens (three types of stitch structure, two directions of the fabric, three water temperature values, five curing ages, with three repetitions) were made, and the tensile properties, absorbed energy, and the inversion effects were evaluated. The results showed that the curing conditions of the reinforced cementitious composite in water with temperature values of 7, 23, and 50 °C affect the tensile behavior. The tensile strength of the CCs cured in water with a temperature of 23 °C had the highest tensile strength, while 7 and 50 °C produced a lower tensile strength. The inversion effect has been observed in CC at 23 °C between 7 and 28 days, while this effect has not occurred in other curing temperature values. By examining three commercial types of stitches in fabrics and the performance of the reinforced cementitious composites in the warp direction, it was found that the structure of the “Tuck Stitch” has higher tensile strength and absorbed energy compared to “Knit stitch” and “Miss Stitch”. The tensile strength and fracture energy of the CC reinforced with “Tuck Stitch” fabric in the warp direction, by curing in 23 °C water for 7 days, were found to be 2.81 MPa and 1.65 × 10^3^ KJ/m^3^, respectively. These results may be helpful in selecting the design and curing parameters for the purposes of maximizing the tensile properties of textile CAC composites.

## 1. Introduction

The development of lightweight composites with high tensile strength is gaining increasing attention by the industry in several sectors due to their versatility for numerous applications. As far as the construction industry is concerned, many composites, such as unreinforced concrete, have good compressive strength and poor tensile strength. However, by adding a reinforcing component, the tensile and flexural properties can be expected to improve. Textile reinforced concrete (TRC) has several advantages: tensile strength, good flexibility, low weight, low thickness, and corrosion resistance [1,2]. These advantages make them very appealing to the market for construction materials. However, their potentialities and limitations have not been fully explored yet, especially when the textile reinforcement is used in combination with special types of cements such as low-emission calcium aluminate cements. For this reason, this study aims to contribute to deepening the knowledge on these types of cementitious composites, on their tensile properties and on the factors that may affect them.

Reinforced cementitious composite with 3D fabric consists of spacer fabric and cementitious material. Spacer fabrics consist of two parallel fabrics connected by spacer yarns. The use of 3D fabrics, due to low weight and high volume, entailed the occupation of a large proportion of the overall cementitious composites, reduced the material consumption and, as a result, significantly reduced the weight of the composites. Reducing the consumption of materials is economical. On the other hand, usually, if too much bending or tensile load is applied, TRC composites will break but will not separate due to the bridging effect of fibers or fabric.

Previous studies have shown that a variety of textiles have effectively reinforced concrete matrices. The use of fibers [3,4], yarns, and fabrics (2D [5,6], 3D [7,8]) are among the most commonly used cementitious mortar reinforcements. Armakan and Roye [9] investigated the use of 3D fabrics to reinforce concrete and the compressive behavior of reinforced concrete. They found that the compressive strength decreased by reducing the angle of placement of the spacer yarns and when reducing the number of these yarns. Munck et al. [10] investigated similar specimens made from a combination of special materials using premix mortar with multiple layers of alkali-resistant (AR) fiberglass fabrics. The obtained results by [10] showed that cracks formed at lower stress levels after being subjected to environmental loading. CC reinforced with 3D fabric with a high strain rate was also investigated [7]. Some studies used an outer reinforcement layer by a fiber-reinforced polymer (FRP) to improve cementitious composites’ tensile and flexural strengths. The high tensile and shear strengths of FRP-reinforced CC compared with the shotcrete offered greater protection against the risk of landslides in complex situations and emergencies. FRP-reinforced CC significantly improved the safety factor due to its high tensile strength [11]. The tensile and flexural behaviors of reinforced CCs were investigated by uniaxial tensile and four-point bending tests in two directions of weft and warp. Experimental results showed that tensile and flexural properties, in particular flexural strength, were significantly improved in reinforced CC samples [12]. Haik et al. [13] investigated the effect of 3D fabric orientation on the flexural properties of CC. They found that the flexural strength of 3D fabric-reinforced composites is more significant in the weft direction. Amzaleg et al. [14] investigated the flexural strength of four-point CC reinforced with 3D warp knitted fabrics and found that the flexural strength and elongation at break of 3D fabric consisting of aramid yarns was greater than 3D polyester fabric. Hui Li et al. [15] investigated the mechanical properties of reinforced cementitious composites with 3D warp knitted fabric. To improve the tensile strength of the composites, they used a modified ultra-high molecular weight polyethylene layer containing carbon nanotubes (CNT). Michael et al. [16] studied the tensile and flexural properties of CC reinforced with 3D fabrics. They found that the spacer yarns did not significantly affect the tensile behavior of TRC and the number of cracks caused by bending.

With regard to the types of cement, most researchers use Portland cement because of its low cost and greater availability. Still, recently, calcium aluminate cement (CAC) has been widespread. CAC is a particular type of cement considered in CC due to its unique properties [15]. In these composites, CAC is used as an adhesive in the mortar instead of ordinary Portland cement (OPC) [17,18]. Research on CAC has increased significantly over the past decades. This interest is due to the many applications of calcium aluminate cement composite (CACC) in various structures such as industrial sidewalks, wastewater treatment plants, tunnels, and oil rigs, the durability of which is of great importance [18,19]. CACC has several apparent advantages over ordinary Portland cement composite (OPCC), including the higher rate of compressive strength development during the setting and curing process, increased wear resistance, resistance to sulfate attacks, and alkaline-silicate reactions [20]. In addition, CAC production results in less CO_2_ emissions than OPC production [20]. At present, CACs are mainly used in refractory cement and building chemical applications, such as quick-setting mortars [21]. According to the results obtained by previous researchers, during the process of setting and curing of CAC mortar, when the “inversion” occurs, the strength of the CAC mortar gradually drops after it initial increased is observed [22,23]. The duration and speed of this process depend on the amount and constitution of the hydrated phase, environment temperature (i.e., curing age and humidity), and the existence of such a phenomenon goes back to the CAC hydration compositions [24,25,26]. However, to balance the benefits of CAC, when the “inversion” phenomenon happens, denser stable hydrates in mortar media are formed with the release of water and consequent formation of porosity, which significantly reduces the mechanical strength [27,28]. CAC releases a lot of heat in the first 24 h of hydration, which can have a significant exothermic effect [29]. The hydration product at low-temperature values (below 29 °C) is CAH_10_ (where C = CaO, A = Al_2_O_3_ and H = H_2_O, according to the notation chemistry of cements), which is thermodynamically unstable. With increasing temperature (between 30 to 70 °C), the product C_2_AH_8_ is semi-stable. At a temperature of more than 70 °C, the product C_3_AH_6_ is stable. Changing the phase from the unstable to the stable one reduces the volume and increases the porosity, thus reducing the strength [21]. In various studies, temperature ranges for phase stability were different; some studies report temperature values below 10 °C for the unstable state, between 10 and 20 °C for the semi-stable one, and temperature values above 30 °C set to the stable phase [23]. In other studies, values below 15 °C led to an unstable phase, between 15 and 30 °C to a semi-stable one, and above 45 °C to a stable one [9,10,11].

Due to the effect of the water-to-cement ratio on the hydration rate [30] and cracking ability [31], many studies have investigated this ratio. The majority of researchers agree that the optimal ratio of water to cement is around 0.4 [30,31,32,33]. Phillips and Zhang [34] found that the compressive strength decreases with increasing water-to-cement ratio because the evaporation of water not used in the hydration process with time leads to porosities in the concrete microstructure. It can ultimately reduce the ultimate compressive strength of concrete [35,36].

As mentioned, cement mortar has low tensile strength and fracture strain; therefore, TRC technology is used to reinforce cement mortar. Due to the spacer yarns, the reinforcement of cement mortar by knitted 3D fabrics has received much attention in the fabric structure. The role of using knitted 3D fabrics with spacer yarns on a smaller scale is similar to the position of using reinforcement to reinforce a concrete structure. Three-dimensional fabric acts as a reinforcing component that significantly improves the tensile strength. In addition to the type of fibers, the structure of the textiles, including yarn density, loop structures [37], 3D fabric thickness, density [38], and the placement angle of monofilament spacer yarns [9,39], count and Young’s modulus of yarns [40] can significantly affect the reinforcement. Hesami et al. [41] found that some energy is absorbed if the loops are easily displaced and deformed by any applied force. According to previous studies, to improve the tensile properties of cementitious composites, it was found that 3D fabrics with CAC mortar help to increase the tensile strength and decrease the setting time. Moreover, it will make the CC usable in less time.

Based on the considerations reported above regarding the potential benefits and possible influencing factors of 3D fabric-reinforced cementitious composites using calcium aluminate cement, the calcium aluminate cement was considered because of its higher strength at an early age. Because the inversion effect is important and unavoidable in calcium aluminate cement, the simultaneous effects of temperature and curing age in CAC mortar are investigated. In this regard, since 3D weft-knitted fabric machines are cheaper and easier to access than 3D warp-knitted fabric machines, therefore, in this study, the 3D weft-knitted fabric was used instead of a warp-knitted one, in addition to the effect of three different hole patterns (stitch structures) of the upper layer of the 3D fabrics in warp and weft directions on the tensile properties of the composite. 

## 2. Materials and Methods

This section details the characteristics of the textile-reinforced cementitious composites analyzed here and the type of tests adopted to study their performances. The final CC properties as 3D fabric-cemented composites depend mainly on 3D fabric cement media and the stitch structure. Therefore, three weft-knitted 3D fabric designs with the same polymeric yarn (Nylon-Polyester) were knitted. Three fabric stitches (tuck stitch, miss stitch, and knit stitch) were used in this experimental work to show the effect of the fabric structures on the tensile strength of the reinforced cementitious composites. For a more precise explanation of the details of the stitch structure and the shape of the holes of the outer layers of the 3D fabric, which is one of the essential components of the cement composite, Table 1 was compiled and is presented in the next section.

### 2.1. Material

**Figure 1:**
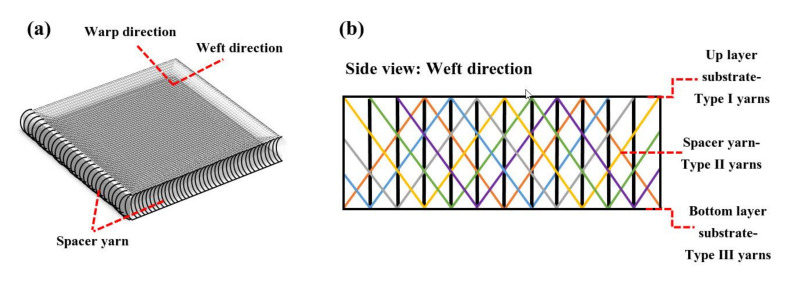
Schematic of 3D spacer fabric (**a**) global view, and (**b**) side view.

In the CCs, a knitted 3D fabric is used to strengthen a CAC-based cementitious matrix. The 3D fabric is considered a reinforcing component divided into the outer substrates and the monofilament spacer yarns that connect the two outer substrates. Figure 1 shows the schematic of this knitted 3D fabric and its components. The fabrics used have different hole patterns in the outer substrate depending on the type of stitch. Accordingly, the Type Ι yarn indicates the upper layer outer substrate, the Type ΙΙ yarn indicates the spacer yarns, and the Type ΙΙΙ yarn indicates the bottom layer outer substrate (Figure 1b).

Three types of knitted 3D fabrics with different stitch patterns in outer layers have been used to investigate the effect of stitch structure on the tensile strength of the CC. The thickness of the fabrics was constant (5 mm) and contributed to reducing the weight of the CC (EN ISO 5084 [42]). The structure parameter and knit pattern of layers of spacer fabrics and the top/bottom layer of the outer textile substrate are shown in Table 1. In all three types of fabric, the top layer of the outer textile substrate has no holes (solid fabric). The stitch structure and, as a result, the hole pattern in the bottom layer of the outer textile substrate is a variable factor in the fabric. In spacer fabric type 1 (SF1), the stitch structure in the bottom layer is “knit stitch”, “miss stitch” in SF2, and “tuck stitch” in SF3. This difference in the type of stitch structure has led to a change in the size and hole patterns (mesh fabric), which this study intends to investigate. The yarns used in the outer substrates are polyester multifilament. The spacer yarns are nylon monofilament with technical specifications of 80/2 Den (0.1 mm).

Different shapes of fabric holes are caused by different stitch structures in the three fabrics, SF1-SF2-SF3. The form of the holes in the fabric changes with the stitch structure of the bottom layer of the 3D fabric and the movement of the knitting needles. In preparing these three types of fabrics, the density of monofilament yarns and the angle of their placement between two outer substrates are constant. Only the texture type of the bottom layer of the fabric is considered a variable factor.

The approximate shapes of the holes in the bottom layer of SF1-SF2-SF3 were rectangular, parallelogram, and without regular geometric shape, respectively. The yarn fineness (Den) of the bottom layer of the fabric (mesh fabric) and the top layer of the fabric (solid fabric) are 150 Den and 150/8 Den, respectively, and the middle yarn fineness is 80/2 Den. 

**Figure 2:**
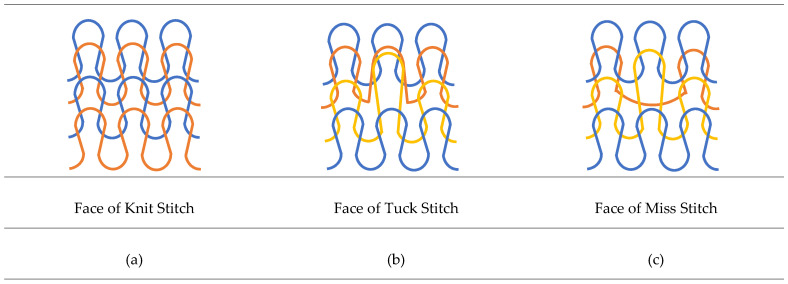
Schematic of 3 types of spacer fabric: (**a**) knit stitch (SF1), (**b**) tuck stitch (SF2), and (**c**) miss stitch (SF3).

In the SF1 fabric, which uses the “knit stitch” loop structure, all the loops were woven. In SF2, which uses a “miss stitch” loop structure, the needle releases the desired yarn and knits another yarn, and this occurs when the needle remains at rest for as long as a knit operation, and then in the next loop performs the knit. In SF3, with a “tuck stitch” loop structure, the needle holds the previous loop on the leg and takes the fed yarn, in which case the last loop does the knitting halfway, but it does not do the second half of the knit, which is releasing the loop, which is why this loop is called a tuck stitch. A schematic of all three types of stitch structures is shown in Figure 2. According to Figure 2, it can be seen that in the form of the “knit stitch” loop, the loops and yarns fed are uniformly located in the whole fabric, while in the structure of the “miss stitch” loop, a floating yarn behind the loop is located. The upper loop is engaged only with its upper loop.

The cement mortar media’s chemical composition includes a combination of silica filler and CAC (according to EN 196-2 [43] and EN 1464 [44]), as shown in Table 2.

### 2.2. Methodology

Different types of CC’s specimens from cement and fabrics with different types of structure and materials were prepared. Since the dry mix must be injected into the 3D fabric without water, the CAC and sand were mixed in a blender for about 10 min with a mixing ratio of 1:3. The dry matrix is poured into the fabrics through the holes of the bottom layer (mesh fabric) and vibrates to make sure it is completely filled. This process of filling the fabric with dry cement matrix is almost similar to Li’s paper [11]. A thin layer of fabric impregnated with a tiny adhesive of hot glue was pressed by heat onto the surface of the bottom layer substrate so that the dry matrix did not spill out of the holes since the water-to-cement ratio was adjusted to 0.366; then, water was sprayed onto the CC surface and was calculated taking into consideration the specimen’s specific area and thickness. After casting, the molds were stored for 6 h at 22 °C and 95% humidity and then cured in water at different ages (i.e., 6 h, 1, 3, 7, and 28 days) with different temperature values (i.e., 7, 23, and 50 °C) before tensile tests. Table 3 presents this research tests setup program. To have the statistically validated data, the three specimens were tested for each mix design. These three temperature values were chosen to investigate the stable, unstable, and semi-stable states mentioned before. The age of 6 h was considered to evaluate the early tensile strength of the CCs. A tensile test was performed for all specimens in both the warp and weft directions of the fabrics.

#### 2.2.1. Yarns and 3D Fabrics Tensile Test

**Figure 3:**
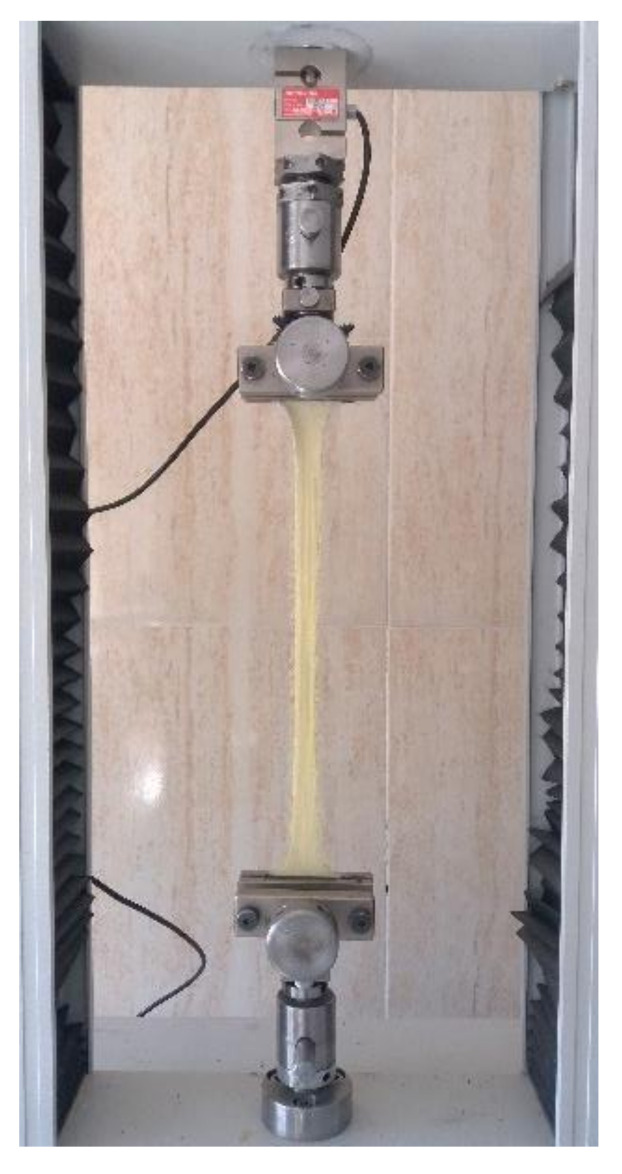
Tensile test of the fabric.

The tensile tests on yarn were performed with a Shirley Micro350 machine with a capacity of 250 KN. The related test speed was 10 mm/min, the distance between the clamps was 500 mm, and the Iranian standard 29 [45] (ISO 2062) was used. The tensile tests on the 3D fabrics were performed with an Instron machine with 1- and 3-ton capacity. The tensile test speed of 3D fabrics was 50 mm/min, the distance between the tensile clamps was 200 mm, and the EN ISO 13934-1 [46] standard was used. The deformation of the fabric under the test is shown in Figure 3. The dimensions of the 3D fabric samples were 5 × 50 × 200 mm^3^. Due to the structural difference of the stitch in the warp and weft direction of the 3D fabrics, a tensile test in each direction was performed separately with three repetitions. All specimens were kept at rest in the laboratory before testing.

#### 2.2.2. Reinforced Cementitious Composites Tensile Test

**Figure 4:**
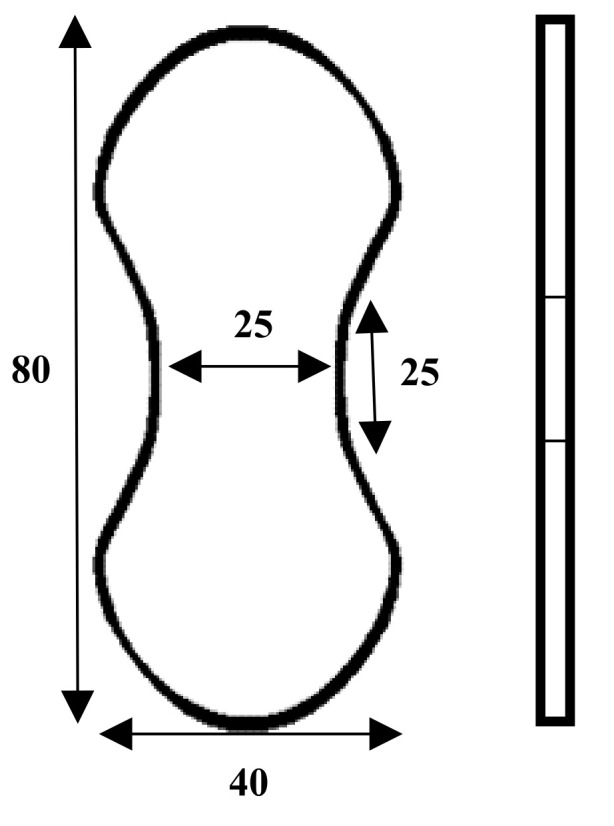
Schematic of dog-bone specimen for the CC tensile test.

According to previous studies and considering the structural differences in the stitch of knitted fabrics in warp and weft directions, it is also necessary to test the tensile behavior of reinforced cementitious composites separately in both directions. The Instron testing machine with a maximum capacity of 3 tons performed the tensile test on the reinforced CCs according to ASTM C307 [47] standard. The test speed was 5 mm/min. The geometry and dimensions of the reinforced cementitious composite specimens used for the uniaxial tensile test are shown in Figure 4. The samples are placed in dumbbell-shaped clamps, and after the start of the test, the tensile load is applied to the CC, and the components of the CC (mortar and fabric) resist this load until the moment of stopping the test. The dumbbell-shaped specimens are almost identical to that of Fakhim’s [48] and Nguyen’s [49] articles. The test stops when the CC breaks or the bottom layer of the outer substrate of the 3D fabric is torn or separated. The mean values of stress and strain of the reinforced cementitious composites were compared in the warp and weft directions. The maximum stress and strain and the stress and strain at the moment of failure were also obtained.

## 3. Results and Discussion

In this section, the results are discussed in four different parts. The first part is focused on the tensile properties of different yarns, and the second investigates the tensile strength of the fabrics of the bottom and the top layer of the 3D fabric. Part 3 investigates the cement mortar properties under tension, and in the final section, the textile-reinforced cementitious composites obtained from the 3D fabric filled with CAC mortar, after curing at different ages, were tested to investigate the effect of temperature and curing ages on the tensile properties of the CCs.

### 3.1. Tensile Properties of Yarn

The results of the different yarn’s (i.e., warp/weft and spacer yarns) tensile tests are summarized in Table 4. The results showed that the tensile strength of the monofilament yarns is the same in all three types of 3D fabrics. In addition, the tensile strength of multifilament yarns forming the top layers of the outer substrate of 3D fabric are the same in all three-fabric types. 

The spacer yarns provide a high stiffness to the 3D fabric. Therefore, by adding cementitious material into the 3D fabric from bottom-layer holes, despite the high weight of cementitious materials, the 3D fabric is not compressed, and its thickness does not decrease. Therefore, there is no space between the cementitious material and the layers of fabric. During the tensile test, the interaction of the tensile capacity of the spacer yarns and the upper/bottom layers of fabric are considered together. Therefore, the difference between the stiffness of the spacer yarns and the upper and bottom layers yarns should not be significant so as not to tear the stitches. Thus, the tensile Young’s modulus of the spacer yarn was about twice that of the yarn of the upper and bottom layers.

**Figure 5:**
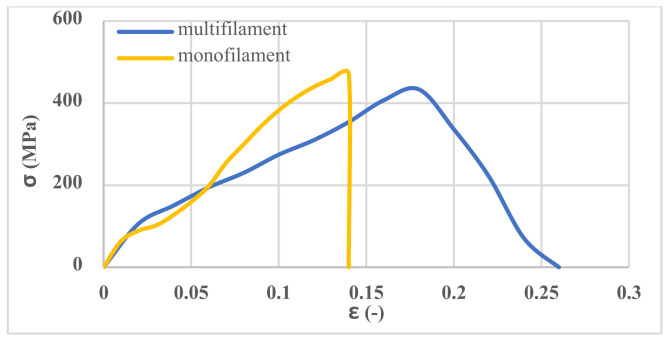
Tensile stress–strain curves of various yarns.

The stress–strain curve for monofilament and multifilament yarns is shown in Figure 5.

### 3.2. Tensile Properties of 3D Fabrics 

**Figure 6:**
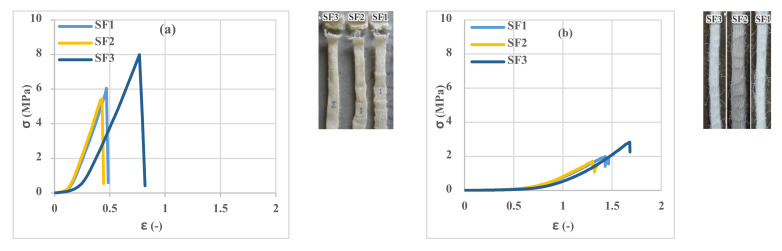
Tensile stress–strain curves of 3D spacer fabrics: (**a**) weft direction and (**b**) warp direction.

The tensile results of 3D fabrics in terms of warp and weft directions are shown in Figure 6. At the beginning of the tensile test, due to the deformation of the loops in the fabric structure and the elongation of the loops, a considerable strain under the pre-load is observed in the fabric stress–strain curves. As the stretching process continued and sustained tensile force by yarns, the width of the fabric samples in the direction perpendicular to the tensile axis decreased, and the force increased. The results showed that the tensile strength of the three types of 3D fabrics is different due to the different stitch structures of the fabrics. As shown in Figure 6, the tensile strength of the SF2 in the warp and weft directions is the lowest, and the SF3 is the highest with an approximately 50% higher value than the SF2. In addition, the fracture and ultimate strain of the SF2 are less than the SF1 and SF3, and therefore, this type of fabric has less energy absorbance than the others. By applying tension force to the fabric in the weft direction and according to the type of connection of the monofilament spacer yarns, these yarns provide less freedom of movement to the fabric, so in a relatively low strain, much stress is applied to the fabric. The fabric is torn from a specific point, usually from the beginning or end of the fabric, and near the tensile test setup clamps. However, a considerable strain was observed by applying tension to the fabric at the warp direction due to the greater freedom for the movement of the monofilament spacer yarns. In the tensile test of the fabric in the warp direction, several weft yarns usually come together, and tear occurs along with the sample. By comparing the first and second parts of Figure 6, it can be concluded that the tensile strength of 3D fabrics in the weft direction is higher than the warp direction. In addition, the fracture and the ultimate strain of 3D fabrics in the warp direction are higher than in the weft direction. Therefore, it can be concluded that the different stitch structures of the fabric influenced the tensile strength of the 3D fabric.

### 3.3. Investigation of Cement Phase Properties in Tension

**Figure 7:**
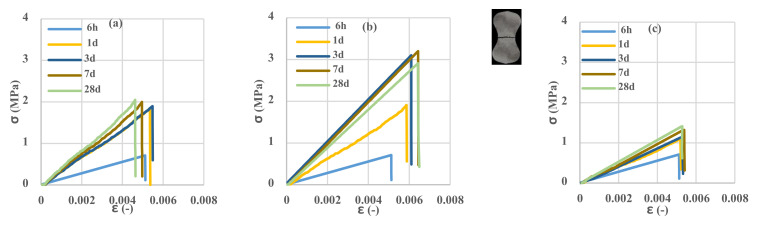
Tensile stress–strain curves of the unreinforced control samples. (**a**) 7 °C, (**b**) 23 °C, (**c**) 50 °C.

To evaluate the influence of the temperature and curing age of cement mortar specimens on the tensile strength, cement mortar samples in standard molds were prepared and tested. The results in Figure 7 show that the specimens are entirely broken with just one crack. It is observed that the trend of changes in the strength of cement mortar at a specific temperature is different so that at 7 and 50 °C, with increasing the age of the samples, tensile strength increases; however, at 23 °C, after 7 days, the process of reaching strength is reversed to 28 days. The phenomenon of inversion is visible at this temperature. According to Juenger’s research [21], at 7 °C, the probability of inversion is much higher than at 23 °C, but due to the decrease in hydration speed, this effect is expected to be visible after 28 days. However, according to various researchers [9,11,23], the increase in temperature is so effective in reducing this phenomenon that this effect was not observed at 50 °C. As shown in the results, the behavior of the cement mortar in terms of maximum tensile strength and fracture strain at 50 °C is lower than the behavior of the mortar at low and medium temperature values. Changing the material structure at high temperature from the unstable phase of CAH_10_ to the stable phase of C_3_AH_6_ has weakened the molecular structure and thus reduced the hardness of the material and the ultimate tensile strength. This result is consistent with the result of Juenger’s research [21].

### 3.4. Tensile Behavior of Reinforced Cementitious Composites

**Figure 8:**
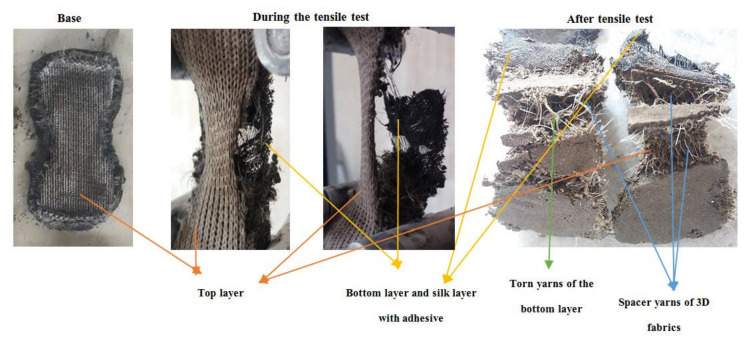
The tensile performance of reinforced cementitious composite.

In this section, the tensile performance of reinforced cementitious composite is presented in Figure 8. Figure 8 shows the CC components reinforced with 3D fabric before, after, and during the tensile test. All the CC parts were stretched at the beginning of the test, and after the test, the top layer of 3D fabric was not torn, but the bottom layer was also torn along with the layer impregnated with the adhesive and the spacer yarns of 3D fabric; this behavior was observed for all reinforced cementitious composites. CCs’ shape changes during the tensile test, depending on the type of stitch structures and the warp or weft direction of the fabric. As a consequence, with respect to unreinforced samples (Figure 7), a strain-hardening behavior was observed in all reinforced samples due to multiple cracking formations.

**Figure 9:**
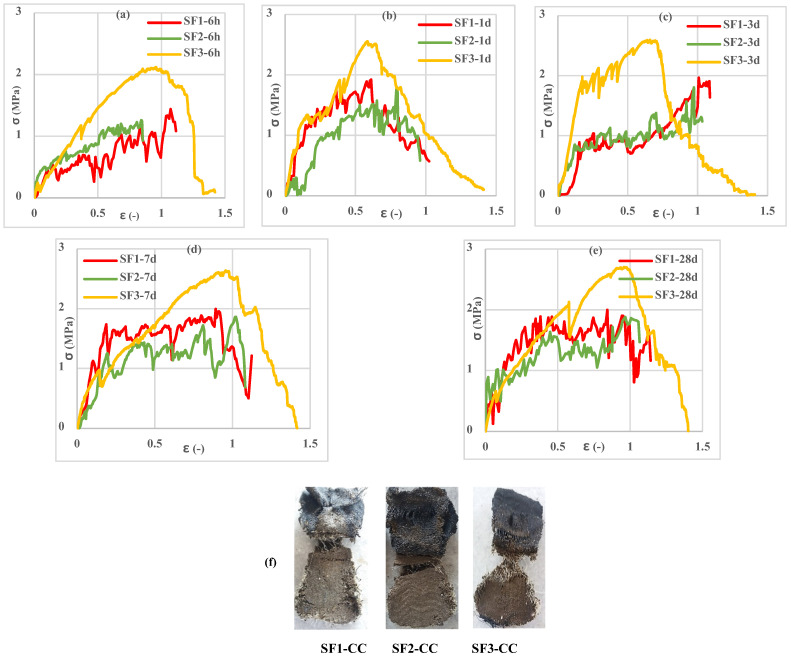
Tensile stress–strain of reinforced mortar with 3 types of spacer fabrics in the warp direction cured at 7 °C temperature (**a**) 6 h, (**b**) 1 d, (**c**) 3 d, (**d**) 7 d, (**e**) 28 d, and (**f**) warp specimens after the tensile test.

**Figure 10:**
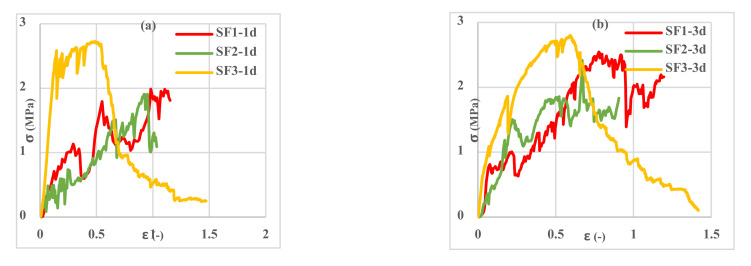

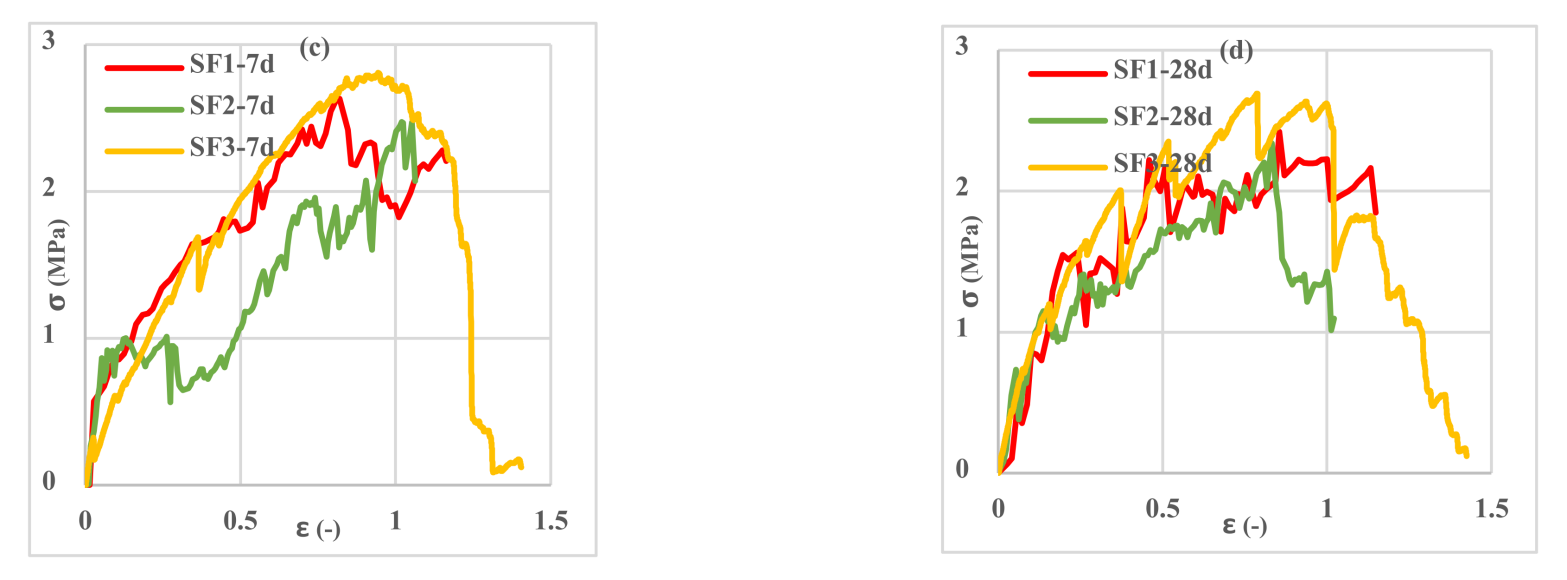
Tensile stress–strain curves of reinforced mortar with 3 types of spacer fabrics in the warp direction cured at 23 °C temperature (**a**) 1 d, (**b**) 3 d, (**c**) 7 d, and (**d**) 28 d.

**Figure 11:**
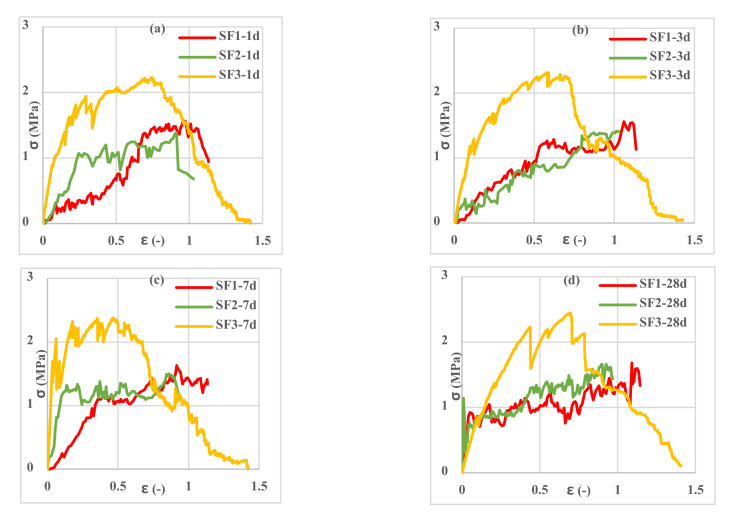
Tensile stress–strain curves of reinforced mortar with 3 types of spacer fabrics in the warp direction cured at 50 °C temperature (**a**) 1 d, (**b**) 3 d, (**c**) 7 d, and (**d**) 28 d.

**Figure 12:**
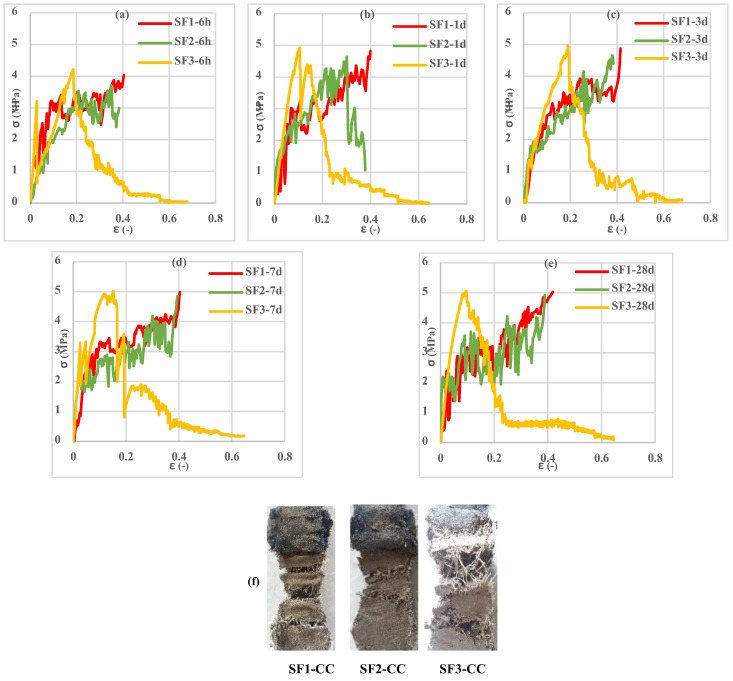
Tensile stress–strain curves of reinforced mortar with 3 types of spacer fabrics in the weft direction cured at 7 °C temperature (**a**) 6 h, (**b**) 1 d, (**c**) 3 d, (**d**) 7 d, (**e**) 28 d, and (**f**) weft specimens after tensile test.

**Figure 13:**
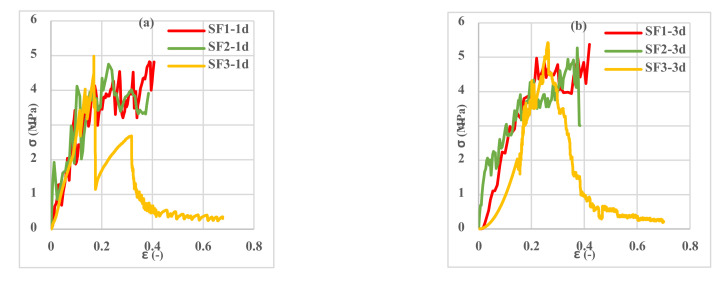

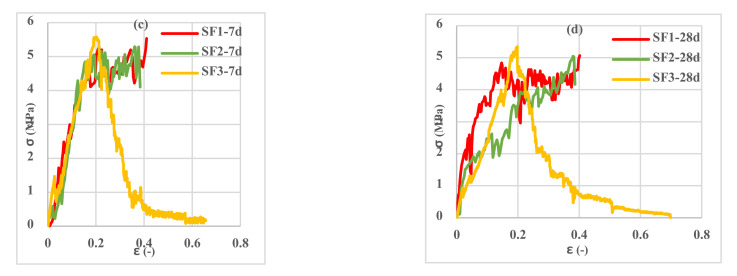
Tensile stress–strain curves of reinforced mortar with 3 types of spacer fabrics in the weft direction cured at 23 °C temperature (**a**) 1 d, (**b**) 3 d, (**c**) 7 d, and (**d**) 28 d.

**Figure 14:**
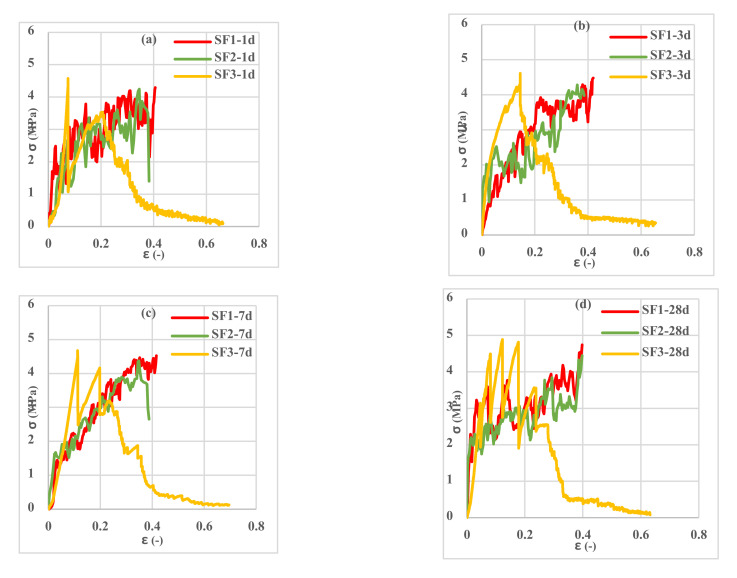
Tensile stress–strain curves of reinforced mortar with 3 types of spacer fabrics in the weft direction cured at 50 °C temperature (**a**) 1 d, (**b**) 3 d, (**c**) 7 d, and (**d**) 28 d.

Investigation of the different spacemen results in Figure 9, Figure 10, Figure 11, Figure 12, Figure 13 and Figure 14 reveals two different fracture types in CC specimens under pure tensile force. First, the mortar initially sustains the applied tensile load up to thorough cracking, and after the mortar breaks, all the applied force will be transferred to the 3D fabric. In this type, only one crack was observed in the middle of the specimens, and in the weft direction, several cracks were observed as a significant fall was observed in the tensile stress–strain graphs. In the second fracture type, the reinforced cementitious composite cracks in both directions, and the specimens’ failure was observed with several cracks. The cracking pattern during the test demonstrated that the tensile force was applied to the stitches of 3D fabric and then it was applied to the yarns. It means that, due to the proper connection between the 3D fabric and the mortar in reinforced cementitious composites, at the same time as the fabric resists the tensile load, the mortar is cracked at different points, and several cracks are observed in the CC. The reinforced cementitious composite is gradually stretched and broken, and in the next steps, the load is then redistributed within the sample and the remaining intact parts are stretched and broken in turn. The fluctuations observed in the different curves of Figure 9, Figure 10, Figure 11, Figure 12, Figure 13 and Figure 14 confirm this multiple crack formation. In addition, As the results in Table 5, the difference between the maximum amount of ultimate stress and fracture stress in most tensile tests of reinforced cementitious composites was precisely related to the stitch type of the bottom layer of the fabric. The results presented that the tensile stresses of the CCs reinforced with SF2 in the warp direction are less than other samples. Furthermore, it can be concluded that multiple cracks patterns are related to the stitch pattern, the mortar, 3D fabric tensile stress, and their effects on each other in the CC.

The results of the tensile test of the fabrics show that they are similar not only in the warp direction but also in the weft direction. In addition, the tensile behavior of reinforced cementitious composites with these fabrics was similar too. By comparing the mortar and CCs test results with each other, it can be concluded that the mortar had a lower tensile strength than the reinforced cementitious composite (i.e., near the 70% more) due to reinforcement caused by the spacer yarns embedded within the mortar.

Figure 9, Figure 10 and Figure 11 show the stress–strain curves of reinforced cementitious composites at 7, 23, and 50 °C in the warp direction, and Figure 12, Figure 13 and Figure 14 in the weft direction, respectively.

According to the types of spacer fabric presented in Figure 2, it can be said that there are no floating yarns in the structure of the “tuck stitch” fabric (SF3), but the middle loop is involved with both the next loop and the upper loop. According to the stitch’s structures, this type of tangled involvement of the yarns increased the tensile strength, the maximum of which is found in the “tuck stitch” fabric structure. In addition, in the weft-knitted fabric with a “tuck stitch” structure, the width of the fabric increases and its elasticity decreases [50], so it can be concluded that the structure of the “tuck stitch” created the maximum tensile strength and maximum elongation. Then, the structure of the “knit stitch” (SF1), due to the homogeneous and uniform distribution of the loops and the homogeneous involvement of the yarns, has the average tensile strength. The “miss stitch” structure (SF2) was due to less involvement and entanglement of the yarns, a floating yarn, and the lowest elasticity [50], which had the lowest tensile strength. In addition, by comparing the tensile strength and strain until failure, it can be said that the CCs reinforced with SF3 had the highest, and then SF1 and finally SF2 had the lowest tensile strength. Thus, it can be concluded that the choice of the “tuck stitch” structure had the highest, then “knit stitch” and finally “miss stitch” had the lowest tensile strength.

By comparing the curves in Figure 9, Figure 10, Figure 11, Figure 12, Figure 13 and Figure 14, it can be concluded that the maximum tensile stress was observed at 23 °C, then at 7 °C, and 50 °C had the lowest tensile stress. This behavior is due to chemical changes in the structure of the CAC mortar and the inversion of the unstable phase to the stable one. 

In all three temperature conditions, it was observed that tensile stress was increased when increasing the curing age of the samples. However, at 23 °C, the tensile stress decreased at 28 days probably due to the inversion phenomenon. In all diagrams, the tensile behavior of CCs reinforced with fabrics SF1 and SF2 has almost the same performance. 

According to the results obtained from the effect of fabric tensile direction (warp or weft) on the tensile stress of the different CCs, the highest tensile strain was observed in the CC tensile in the warp direction due to greater freedom of movement in monofilament yarns.

**Figure 15:**
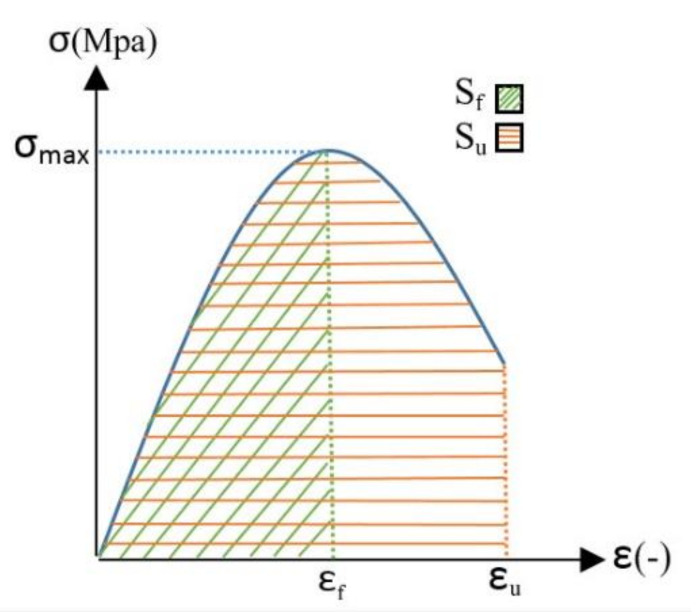
Schematic of stress–strain curve and related relevant quantities.

According to the results presented in Figure 6, SF1 and SF2 fabrics bore lower stress and strain than the SF3 fabric, so when SF1 and SF2 fabrics were used to reinforce the CC, the related specimens failed during the tensile test. In CCs reinforced with SF3, because the bottom layer of fabric and the middle spacer yarns do not tear simultaneously, the stress–strain curve gradually decreases and continues to a minimum at the end of the test. Therefore, the endpoint of the test in the tensile stress–strain curve of SF1 and SF2-reinforced cementitious composites appears in the region with higher stress and lower strain than for SF3-reinforced cementitious composites. According to Figure 15, the strain at the maximum stress is referred to as the “fracture” strain (ε_f_). The strain at the end of the test is denoted as “ultimate” strain (ε_u_), and the ratio of fracture strain to ultimate strain is denoted as cross-sectional strain capacity. The above-defined quantities are reported in Table 6.

The force–displacement curve integral was used to calculate the fracture energy of reinforced cementitious composites. The absorbed energy per unit area was calculated by integrating the area below the curve [51,52]. Figure 15 shows a schematic of a stress–strain curve, with indication of the fracture strain, the ultimate strain, and the subtended area. The energy absorbed per unit area in tensile tests of reinforced cementitious composites at the different temperature values and ages are reported in Table 6. The samples examined in Table 6 are denoted with an abbreviation code in the form of “fabric type—fabric direction—storage age—storage temperature”. For example, the abbreviation code “7 °C-6 h-w SF1” indicates a CC reinforced with SF1 fabric in the direction of width and age of 6 h, stored at 7 °C. 

**Figure 16:**
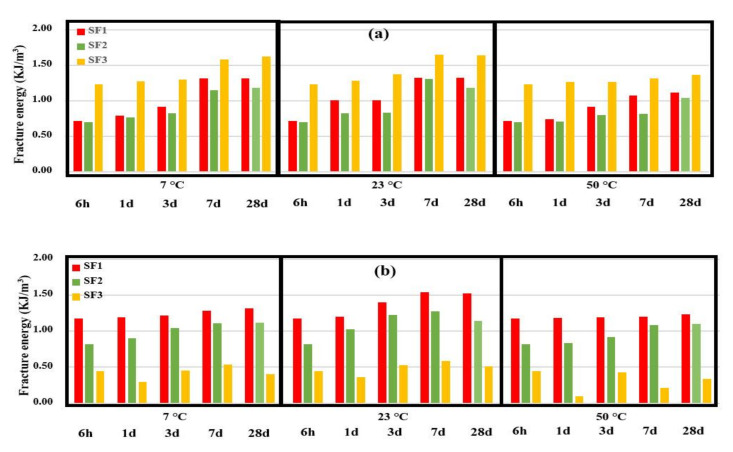
Fracture energy of reinforced CC with spacer fabrics, (**a**) warp direction, and (**b**) weft direction up to ε_f_.

According to Table 6, it was observed that the CC reinforced with SF3 had the highest final strain (ε_u_), then SF1 and finally SF2 had the lowest final strain regardless of the ages, curing temperature values, and different directions of the fabric. These results are also consistent with the fabric tension results (presented in Figure 6). The table above also examines the cross-sectional strain capacity (ε_f_/ε_u_) in different fabric directions. The cross-sectional strain capacity in the weft direction (for all ages and temperature values) for CCs reinforced with different fabrics is as follows: SF1 = 1, 0.59 < SF2 < 1 and 0.11 < SF3 < 0.37. It can be mentioned that the cross-sectional strain capacity was the highest for SF1 fabric-reinforced cementitious composites, followed by SF2 and finally SF3. On the other hand, the cross-sectional strain capacity in the warp direction (for all ages and temperature values) is highest for SF2 fabric-reinforced cementitious composites (0.7 < ε_f_/ε_u_ < 0.9), followed by SF1 (0.5 < ε_f_/ε_u_ < 0.9), and SF3 (0.3 < ε_f_/ε_u_ < 0.6) was the lowest value. The results related to the absorbed energy up to the fracture strain point (maximum tensile stress) are presented in Figure 16.

According to Figure 16, it can be seen that with increasing the curing age of 3D fabric-reinforced cementitious composites, the absorbed energy increased. At the age of 28 days and at 23 °C, due to the inversion phenomenon, tensile stress decreased and absorbed energy reduced. The increasing trend of energy absorption in the different CCs is due to the hardening of the cement phase and to the higher interaction of the cementitious mortar with the fabric. All CCs had this trend, but SF3-reinforced cementitious composites’ absorbed energy did not have a specific trend in the weft direction. The highest fracture energy was determined with the samples cured at 23 °C, then 7 °C, while the samples cured at 50 °C that had the lowest level fracture energy. In 3D fabric-reinforced cementitious composites in the warp direction, SF3 had the highest absorbed energy, and SF1 and SF2 had the lowest absorbed energy. These results are in line with the results of the values of tensile strength. The tensile test of reinforced cementitious composite in the weft direction showed that SF1 had the highest absorbed energy, and SF2 and SF3 had the lowest absorbed energy, which contrasts the results of CCs in the warp direction.

## 4. Conclusions

This research aims to study simultaneous effects of temperature and curing age in CAC mortar and the three different hole patterns (stitch structures) of the bottom layer of the 3D fabrics in warp and weft directions on the tensile properties of the composite. Tensile tests have been performed to obtain warp and weft tensile stress–strain curves, and the fracture energy of the CC has been presented. To investigate and analyze the tensile behavior of reinforced cementitious composite, each of its components, including yarns, 3D fabrics, cement phase, and finally, reinforced composites, were subjected to tensile tests, and the behavior of each was examined separately. Three 3D fabrics with different stitch structures use in the bottom layer, and their effect on the tensile behavior of the CC was investigated. The stress-strain curves of the CCs in different directions (warp and weft), ages, and curing conditions were obtained, and the experimental results were analyzed. The results are presented as follows:

- Three types of 3D fabric (SF1-SF2-SF3) with three different types of loop textures in the bottom layer of the fabric in terms of length and width were stretched, and the SF3 fabric had the highest tensile capacity and ductility compared to the other 3D fabrics. The SF1 had the least amount of stress and strain.
- By examining the amount of stress and strain in fabrics in both warp and weft directions, it was observed that reinforced CCs had higher tensile strength and lower strain in the weft direction than in the warp direction. This trend was observed in all three types of fabrics.

Reinforced cementitious composites with 3D fabrics were studied on the basis of two features: the maximum tensile stress and the maximum absorbed energy. Examination of the ultimate tensile stress of reinforced cementitious CCs showed that the composites reinforced with SF3 have the highest tensile stress; then, CC reinforced with SF1 and reinforced with SF2 fabric. This trend was similar in terms of warp and weft direction. By examining the absorbed energy of reinforced cementitious composites, it was observed that the CCs reinforced with SF3 had the highest absorbed energy in the warp direction. Then SF1 and SF2 had the lowest absorbed energy. CCs reinforced with SF1 had the highest absorbed energy in the weft direction, and then SF2 while SF3 had the lowest absorbed energy.

In this paper, the cement phase mixing design was considered fixed, and the method of achieving this mixing design is presented in a separate article. Different angles of spacer yarns in 3D fabric, yarn material, 3D fabric thickness, etc., could be influential factors that have not been studied in this article, and each can be examined separately in several articles. In this paper, reinforced cement composites were only tested for tensile strength. The results of composite bending tests along with their modeling and optimization by ABAQUS software will be present in future papers.

## Figures and Tables

**Table 1:** Structure parameters and view of 3D spacer fabrics.

3D Spacer Fabric	Density (kg/m^3^)	Amount of Spacer Yarn (Spacer Yarns/cm^2^)	Bottom Layer of Outer Textile Substrate	The Knit Repeat of Layers of Spacer Fabrics		Top Layer of Outer Textile Substrate
				Top Layer	Bottom Layer	
SF1	104.687	63	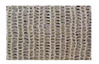	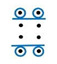	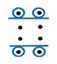	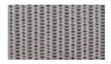
SF2	102.864	80	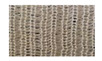	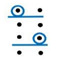	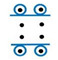	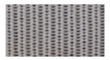
SF3	97.1354	54	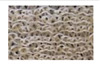	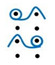	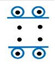	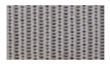

**Table 2:** Chemical compositions of cement mortar media.

Chemical Composition	Cement (%)	Sand (%)
Al_2_O_3_	37	0.29
Si_2_O	4	98.63
CaO	37	0.3
Fe_2_O_3_	15	0.041
Ti_2_O	5	-
MgO	2	0.05
Na_2_O	-	0.02
K_2_O	-	0.07
L.O.I	-	0.5

**Table 3:** Mixing proportions and tests set up program.

Mixing Proportions and Tests Set Up	
sand	1500 kg/m^3^
cement	500 kg/m^3^
cement-to-sand ratio	1:3
water-to-cement ratio	0.366
curing temperature conditions	7 °C, 23 °C, and 50 °C
curing age conditions	6 h, 1,3,7, and 28 days
stitch structures	SF1, SF2, and SF3
tensile test directions	warp and weft

**Table 4:** Properties and geometries of various yarns of different 3D spacer fabrics.

3D Spacer Fabric	Component	Young’s Modulus (GPa)	Tensile Strength (MPa)	Ultimate Elongation (%)	Fineness (den)	Diameter (mm)
**SF1–SF2–SF3**	**Warp/Weft Yarn**	1.712	426.194	25.2	150	0.12
	**Spacer Yarn**	3.328	475.831	14.2	80	0.1

**Table 5:** Tensile stress/strain fracture/ultimate of spacer fabrics at 23 °C, 7 days.

	SF1/Weft	SF1/Warp	SF2/Weft	SF2/Warp	SF3/Weft	SF3/Warp
Tensile stress max (MPa)	5.53	2.6	5.29	2.47	5.57	2.8
Fracture tensile strain (ε_f_)	0.41	0.81	0.36	1.01	0.2	0.94
Ultimate tensile stress (MPa)	5.53	2.2	4.1	2.07	0.18	0.12
Ultimate tensile strain (ε_u_)	0.41	1.16	0.38	1.06	0.65	1.4

**Table 6:** Calculation of fracture strain (ε_f_), ultimate strain (ε_u_), cross-sectional strain (ε_f_/ε_u_), energy absorption until max stress (S_f_) and end of the test (S_u_) of reinforcement CCs at warp and weft directions.

Weft Direction	ε_f_	ε_u_	ε_f/_ε_u_	S_f_	S_u_	Warp Direction	ε_f_	ε_u_	ε_f/_ε_u_	S_f_	S_u_
**7C-6h-w SF1**	0.404	0.404	1.000	1.175	1.175	7C-6h-C SF1	1.072	1.115	0.962	0.715	0.769
**7C-6h-w SF2**	0.349	0.383	0.910	0.822	0.920	7C-6h-C SF2	0.841	0.853	0.986	0.694	0.708
**7C-6h-w SF3**	0.186	0.677	0.275	0.444	0.833	7C-6h-C SF3	0.955	1.421	0.672	1.229	1.801
**7C-1d-w SF1**	0.400	0.400	1.000	1.196	1.196	7C-1d-C SF1	0.612	1.024	0.597	0.787	1.223
**7C-1d-w SF2**	0.302	0.379	0.798	0.900	1.071	7C-1d-C SF2	0.795	0.960	0.828	0.763	0.946
**7C-1d-w SF3**	0.106	0.642	0.165	0.297	0.858	7C-1d-C SF3	0.586	1.412	0.415	1.275	1.840
**7C-3d-w SF1**	0.415	0.415	1.000	1.219	1.219	7C-3d-C SF1	1.007	1.088	0.926	0.917	1.317
**7C-3d-w SF2**	0.380	0.385	0.987	1.049	1.072	7C-3d-C SF2	0.973	1.033	0.942	0.821	0.994
**7C-3d-w SF3**	0.189	0.679	0.279	0.452	0.981	7C-3d-C SF3	0.659	1.409	0.468	1.300	1.855
**7C-7d-w SF1**	0.403	0.403	1.000	1.285	1.285	7C-7d-C SF1	0.892	1.124	0.794	1.312	1.591
**7C-7d-w SF2**	0.394	0.394	1.000	1.108	1.108	7C-7d-C SF2	1.018	1.082	0.941	1.149	1.243
**7C-7d-w SF3**	0.153	0.646	0.237	0.534	1.024	7C-7d-C SF3	0.957	1.416	0.676	1.581	2.254
**7C-28d-w SF1**	0.419	0.419	1.000	1.322	1.322	7C-28d-C SF1	0.842	1.142	0.738	1.319	1.640
**7C-28d-w SF2**	0.389	0.389	1.000	1.115	1.115	7C-28d-C SF2	0.966	1.065	0.906	1.180	1.360
**7C-28d-w SF3**	0.095	0.646	0.148	0.408	1.035	7C-28d-C SF3	0.958	1.401	0.684	1.621	2.114
**23C-1d-w SF1**	0.388	0.406	0.955	1.200	1.280	23C-1d-C SF1	0.982	1.153	0.851	1.003	1.323
**23C-1d-w SF2**	0.227	0.384	0.591	1.024	1.230	23C-1d-C SF2	0.923	1.035	0.892	0.822	0.979
**23C-1d-w SF3**	0.169	0.677	0.250	0.365	0.875	23C-1d-C SF3	0.488	1.472	0.331	1.280	1.867
**23C-3d-w SF1**	0.420	0.420	1.000	1.399	1.399	23C-3d-C SF1	0.778	1.195	0.651	1.008	1.885
**23C-3d-w SF2**	0.373	0.383	0.973	1.235	1.275	23C-3d-C SF2	0.670	0.907	0.739	0.832	1.225
**23C-3d-w SF3**	0.263	0.700	0.375	0.533	1.042	23C-3d-C SF3	0.594	1.416	0.420	1.373	2.026
**23C-7d-w SF1**	0.410	0.410	1.000	1.540	1.540	23C-7d-C SF1	0.819	1.162	0.705	1.327	2.064
**23C-7d-w SF2**	0.360	0.384	0.939	1.276	1.388	23C-7d-C SF2	1.019	1.063	0.959	1.307	1.409
**23C-7d-w SF3**	0.203	0.656	0.309	0.586	1.151	23C-7d-C SF3	0.944	1.404	0.672	1.652	2.400
**23C-28d-w SF1**	0.402	0.402	1.000	1.523	1.523	23C-28d-C SF1	0.855	1.148	0.745	1.321	1.941
**23C-28d-w SF2**	0.380	0.387	0.983	1.148	1.180	23C-28d-C SF2	0.833	1.021	0.815	1.186	1.390
**23C-28d-w SF3**	0.198	0.968	0.205	0.513	1.049	23C-28d-C SF3	0.788	1.425	0.553	1.638	2.396
**50C-1d-w SF1**	0.406	0.406	1.000	1.184	1.184	50C-1d-C SF1	0.973	1.131	0.860	0.743	0.950
**50C-1d-w SF2**	0.345	0.382	0.902	0.840	0.973	50C-1d-C SF2	0.913	1.029	0.887	0.710	0.939
**50C-1d-w SF3**	0.075	0.663	0.113	0.098	0.844	50C-1d-C SF3	0.745	1.418	0.525	1.265	1.810
**50C-3d-w SF1**	0.420	0.420	1.000	1.194	1.194	50C-3d-C SF1	1.060	1.136	0.933	0.914	1.026
**50C-3d-w SF2**	0.359	0.385	0.934	0.918	1.021	50C-3d-C SF2	1.013	1.030	0.984	0.794	0.952
**50C-3d-w SF3**	0.145	0.654	0.221	0.427	0.965	50C-3d-C SF3	0.581	1.428	0.406	1.270	1.589
**50C-7d-w SF1**	0.416	0.416	1.000	1.205	1.220	50C-7d-C SF1	0.917	1.135	0.807	1.074	1.140
**50C-7d-w SF2**	0.348	0.387	0.899	1.088	1.085	50C-7d-C SF2	0.862	0.960	0.898	0.812	1.099
**50C-7d-w SF3**	0.113	0.695	0.162	0.215	0.991	50C-7d-C SF3	0.467	1.422	0.328	1.318	1.612
**50C-28d-w SF1**	0.398	0.398	1.000	1.237	1.237	50C-28d-C SF1	1.092	1.144	0.955	1.117	1.195
**50C-28d-w SF2**	0.397	0.397	1.000	1.103	1.103	50C-28d-C SF2	0.926	0.969	0.956	1.019	1.110
**50C-28d-w SF3**	0.123	0.632	0.194	0.342	1.024	50C-28d-C SF3	0.696	1.408	0.495	1.369	1.921

## Data Availability

Not applicable.
